# Key Transport and Ammonia Recycling Genes Involved in Aphid Symbiosis Respond to Host-Plant Specialization

**DOI:** 10.1534/g3.118.200297

**Published:** 2018-05-16

**Authors:** Dohyup Kim, Bushra F. Minhas, Hongmei Li-Byarlay, Allison K. Hansen

**Affiliations:** * Department of Entomology, University of Illinois at Urbana-Champaign, Urbana, IL 61801; † Illinois Informatics Institute, University of Illinois at Urbana-Champaign, Urbana, IL 61801; ‡ Department of Natural Sciences, Central State University, Wilberforce, OH 45384

**Keywords:** DNA methylation, aphid-*Buchnera* symbiosis, insect-plant interactions, GS/GOGAT cycle, transporter *ApGLNT1*, glycine metabolism

## Abstract

Microbes are known to influence insect-plant interactions; however, it is unclear if host-plant diet influences the regulation of nutritional insect symbioses. The pea aphid, *Acyrthosiphon pisum*, requires its nutritional endosymbiont, *Buchnera*, for the production of essential amino acids. We hypothesize that key aphid genes that regulate the nutritional symbioses respond to host-plant diet when aphids feed on a specialized (alfalfa) compared to a universal host-plant diet (fava), which vary in amino acid profiles. Using RNA-Seq and whole genome bisulfite sequencing, we measured gene expression and DNA methylation profiles for such genes when aphids fed on either their specialized or universal host-plant diets. Our results reveal that when aphids feed on their specialized host-plant they significantly up-regulate and/or hypo-methylate key aphid genes in bacteriocytes related to the amino acid metabolism, including glutamine synthetase in the GOGAT cycle that recycles ammonia into glutamine and the glutamine transporter *ApGLNT1*. Moreover, regardless of what host-plant aphids feed on we observed significant up-regulation and differential methylation of key genes involved in the amino acid metabolism and the glycine/serine metabolism, a metabolic program observed in proliferating cancer cells potentially to combat oxidative stress. Based on our results, we suggest that this regulatory response of key symbiosis genes in bacteriocytes allows aphids to feed on a suboptimal host-plant that they specialize on.

When an organism symbiotically lives inside another organism’s cells its cellular metabolic processes often become integrated with its hosts’. Archetypes of these ancient cellular integration events are readily observed in eukaryotic cells as mitochondria and plastids (Dyall *et al.* 2004). Such organelles exhibit complex regulatory mechanisms that control all aspects of cellular processes such as cell division, transport, and metabolism. Similar to the regulation of organelles, simultaneous inter-domain crosstalk of animal host and bacteria exists between host cells and microbial endosymbionts (Zientz *et al.* 2004). This crosstalk is essential to orchestrate the metabolic needs of both players in the symbiosis. One of the clearest examples of these metabolic integration events can be found within intracellular insect-microbe symbioses (Hansen and Moran 2014). More than 10% of insect species possess long-term, mutualistic bacteria that provision nutrients to their insect host, and are housed inside of specialized host cells, referred to as bacteriocytes (Sudakaran *et al.* 2017). Bacteriocytes are adapted to facilitate inter-domain molecular interactions; however, the mechanisms that the host cell uses to regulate, respond to, and control this integrated, symbiotic metabolism is still largely unexplored.

The mutualistic interaction between the pea aphid (*Acyrthosiphon pisum*) and its bacterial endosymbiont, *Buchnera aphidicola*, is one of the best-studied models on nutritional symbioses. In this symbiosis, amino acid pathways of both players are integrated together for the production of essential amino acids (Nakabachi *et al.* 2005; Wilson *et al.* 2010; Hansen and Moran 2011; Poliakov *et al.* 2011). This integrated mixed-domain metabolism ultimately enables aphids to utilize nutrient deficient plant sap as food because like most animals, essential amino acid pathways are not encoded in the aphid’s genome (International Aphid Genomics Consortium 2010). For example, the aphid provides nonessential amino acid inputs to *Buchnera*’s essential amino acid pathways, and then *Buchnera* provides essential amino acids and vitamins to its host (Shigenobu *et al.* 2000; Nakabachi *et al.* 2005; International Aphid Genomics Consortium 2010). Several aphid genes, including genes that recycle ammonia into glutamate, complement *Buchnera*’s essential amino acid pathways, and transport non-essential amino acid inputs into bacteriocytes, are predicted to be key aphid genes involved in the regulation of this nutritional symbiosis (Nakabachi *et al.* 2005; Hansen and Moran 2011; Poliakov *et al.* 2011; Price *et al.* 2014).

Previous research on this system suggests that when dietary amino acid contents vary, aphids and *Buchnera* collectively adjust amino acid biosynthesis based on the aphid’s nutritional requirements (Liadouze *et al.* 1995; Febvay *et al.* 1999). Moreover, when aphids feed on an artificial diet that varies only in nonessential amino acid profiles, bacteriocytes rebuild distinct profiles of amino acids that depend on the initial nonessential amino acid input(s) (Haribal and Jander 2015). Together, these results suggest that bacteriocytes respond to amino acid variation in the aphid’s diet. Host-plants that pea aphids feed upon in the family *Fabaceae* vary dramatically in free amino acid profiles (Sandström and Pettersson 1994). In turn, it is unclear how aphid bacteriocytes regulate their key symbiotic genes within the amino acid metabolism and in other cellular processes when a polyphagous pea aphid line feeds on their specialized host-plant compared to other host-plants (Hansen and Moran 2014), which vary in free amino acid profiles (Sandström and Pettersson 1994).

Eukaryotic regulons are complex and are orchestrated through a combination of multiple mechanisms, including transcription factors, noncoding RNAs, and epigenetic factors. Among these different layers of gene regulation, the importance of epigenetic factors in influencing gene expression and alternative splicing has only recently begun to be elucidated (Luco *et al.* 2011; Romanoski *et al.* 2015). Previously, it has been shown that signals from the environment such as anxiety, stress, and diet can modify DNA methylation, which can subsequently alter gene expression profiles between different tissue types and throughout an organism’s development (Feil and Fraga 2012; Tammen *et al.* 2013). For example, diet can modify DNA methylation patterns in a diversity of animals including insects, which in turn affects gene expression and subsequently influences organismal phenotypes (Niculescu and Zeisel 2002; Kucharski *et al.* 2008; Anderson *et al.* 2012). The pea aphid is an ideal insect to observe methylation patterns in because it possesses an asexual, clonal, parthenogenetic life stage (Dixon 1977) with a functional DNA methylation system (Walsh *et al.* 2010; Pasquier *et al.* 2014; Mukherjee and Baudach 2016). Such variation at the epigenomic level but not DNA level within a clonal aphid population may be advantageous if it leads to transient phenotypes that are associated with dynamic ecological factors such as host-plant nutrition. Currently it is unknown if the pea aphid has differential methylation in different tissue types and if host-plant environment influences methylation patterns in bacteriocytes. Therefore, DNA methylation may play an important role in the regulation of the aphid-*Buchnera* integrated metabolism, especially in response to different nutritional environments.

Here, using RNA-Seq and whole genome bisulfite sequencing we investigate if key aphid genes involved in the regulation of the aphid-*Buchnera* symbiosis are differentially expressed and methylated between a specialized and a universal host-plant diet (*i.e.*, a host-plant diet all aphid biotypes can perform well on). If aphid bacteriocytes can alter these key symbiotic genes in response to feeding on different host-plant environments, aphids can potentially optimize and/or compensate for specialized plant diets that are otherwise unsuitable in nutrient profiles.

## Materials And Methods

See *SI Materials and Methods* for details.

*Acyrthosiphon pisum* strain (LSR1) was divided into 6 independent sub-lines on either fava (F1, F2, and F3) or alfalfa (A1, A2, and A3) and reared at the same conditions as described in Hansen and Moran (2011). For surrogate aphid fitness trials, weights were measured similar to Vogel and Moran (2011) (N = 20 adult individuals per sub-line). *Buchnera* abundance was measured per aphid individual with Real Time quantitative PCR (RT-qPCR) (N= 6 individuals per life stage per sub-line) along with transmission electron microscopy (TEM) (N = 7 bacteriocytes for fava; N = 5 bacteriocytes for alfalfa). Bacteriocytes of each sub-line were dissected and counted using a light microscope.

For RNA-Seq trials, for the same 6 sub-lines pea aphid bacteriocytes and body cells were dissected, and RNA extractions were conducted similar to Hansen and Moran (2011), except RNA > 200 bp was retained for sequencing using HiSeq2500 (Illumina, San Diego, CA). For whole genome bisulfite sequencing, the same 6 sub-lines were dissected, extracted, bisulfite converted, and sequenced in a strand-specific manner on HiSeq2500 (Illumina, San Diego, CA) using a TruSeq SBS sequencing kit (Illumina).

For both host-plant treatments bacteriocyte gene expression was compared to body cell gene expression in this study similar to Nakabachi *et al.* (2005), Hansen and Moran (2011), and Poliakov *et al.* (2011) to identify unique bacteriocyte-specific signatures of gene expression relative to other cell types, regardless of host-plant treatment (BAC *vs.* BODY). To identify host-plant specific differences in bacteriocyte gene expression we compared bacteriocytes of alfalfa feeding aphids (ABAC) to bacteriocytes of fava (FBAC) feeding aphids (ABAC *vs.* FBAC). Specifically, RNA-Seq paired-end data were mapped and analyzed using the HISAT2 (Pertea *et al.* 2016) and StringTie (Pertea *et al.* 2015). Differentially expressed genes for BAC *vs.* BODY were identified using the likelihood ratio tests (LRT) based on generalized linear models with DESeq2 (Love *et al.* 2014) (see *SI Materials and Methods* for details). Differentially expressed genes for ABAC *vs.* FBAC were identified using LRT (see *SI Materials and Methods* for details). Statistical significance of differentially expressed genes were determined with FDR adjusted *P* ≤ 0.05 and ≥1.5X fold change of the normalized expression values.

The HISAT2 (Pertea *et al.* 2015) and DEXSeq (Anders *et al.* 2012) pipelines were used to find differentially spliced genes. Genes with differential exon usages between bacteriocytes and body cells (BAC *vs.* BODY) as well as between bacteriocytes of alfalfa feeding aphids and fava feeding aphids (ABAC *vs.* FBAC) were identified using a generalized linear model-based approach with FDR adjusted *P* ≤ 0.05 (see *SI Materials and Methods* for details).

Similar to above for expression data, bacteriocyte methylation data were also compared to body tissues regardless of host-plant type (BAC *vs.* BODY), and between bacteriocytes from aphids feeding on alfalfa and fava (ABAC *vs.* FBAC). Methylation read data were aligned with Bowtie2 and Bismark, as suggested in Krueger and Andrews (2011). Site-specific CpG methylation data for each sample were calculated using the methylKit package in R with the minimum coverage of 10 reads per site (see *SI Materials and Methods* for details) (Akalin *et al.* 2012). Differentially methylated CpG sites were obtained from methylSig (Park *et al.* 2014). Statistical significance of differentially methylated CpG sites was determined with q ≤ 0.01 and ≥10% methylation differences. GSEA (Subramanian *et al.2005*) was used to determine which KEGG pathways were differentially regulated at the normalized *P* < 0.05 and <0.1 (See *SI Materials and Methods* for details).

### Data availability

The sequence data for directional RNA-Seq and whole genome bisulfite sequencing for all aphid samples were submitted to NCBI under study accession number PRJNA213008 and PRJNA339317, respectively. Supplemental material available at Figshare: https://doi.org/10.25387/g3.6110726.

## Results

In this study, the pea aphid strain (LSR1), which originated as an alfalfa (*Medicago sativa*) specialist in the field (International Aphid Genomics Consortium 2010), was used for all trials. Here, the LSR1 strain was divided into six independent sub-lines for all host-plant trials; three sub-lines fed on its specialized host-plant, alfalfa, and the remaining three sub-lines fed on its ’universal’ host-plant, fava. The universal host-plant fava was chosen because previous studies have indicated that most pea aphid biotypes favor and display higher fitness on their universal host-plant ’fava’ (*Vicia faba*) compared to the host-plant they specialize on in the field (Ferrari *et al.* 2008; Ferrari *et al.* 2012; Peccoud *et al.* 2014). During all trials host-plants were of a particular developmental stage where amino acid profiles in sap vary significantly in alfalfa, compared to fava (Sandström and Pettersson 1994) (see *SI Materials and Methods*).

### Effects of host-plant diet on aphid and Buchnera phenotype

**Figure 1:**
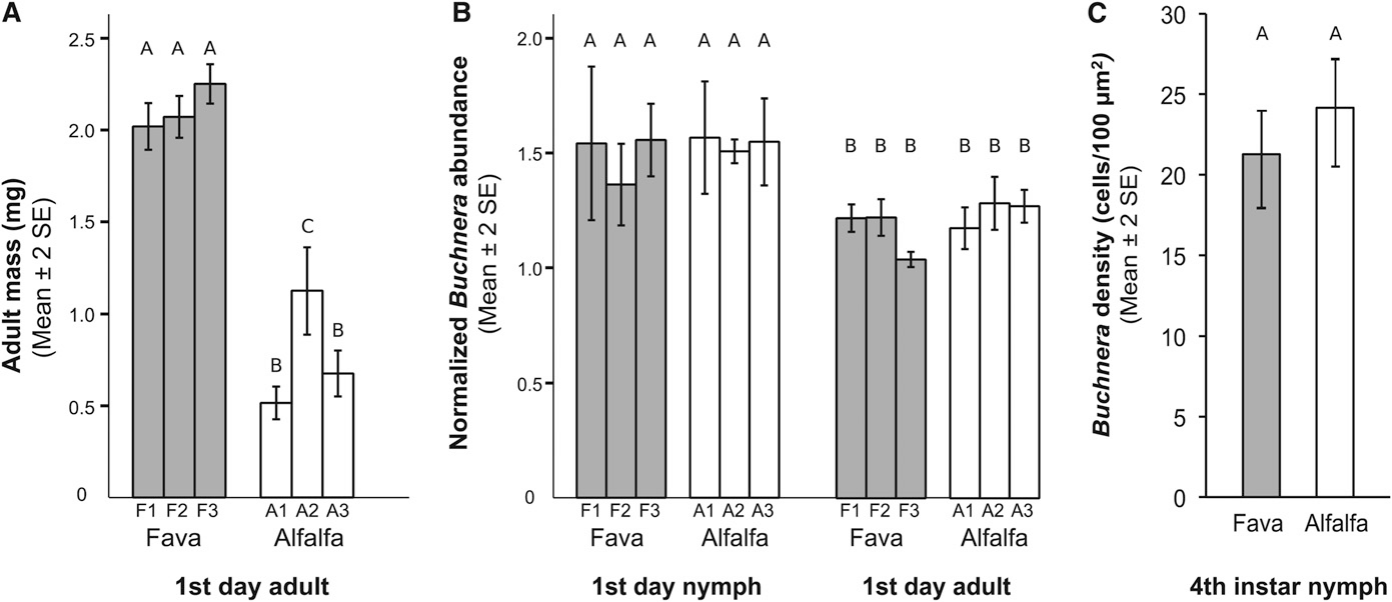
Effects of a specialized host-plant diet on aphid and *Buchnera* phenotype. Different letters above bars indicate significant differences between aphid sub-lines within each sub-figure (A, B, C) (Tukey’s multiple comparison post-hoc test *P* < 0.05) (A) Aphid mass of 1^st^ day adults. N = 20 aphid individuals per aphid sub-line. (B) *Buchnera* abundance of 1^st^ day nymphs and 1^st^ day adults measured by a single copy *Buchnera* gene with RT-qPCR and normalized by a single copy aphid gene. N = 6 aphid individuals per aphid sub-line (C) *Buchnera* cell density of 4^th^ instar nymphs measured by the number of *Buchnera* cells per unit area (100 µm^2^) using TEM.

To investigate if pea aphid LSR1 fitness was significantly greater in aphids feeding on fava compared to their specialist host-plant alfalfa we measured adult aphid mass, a surrogate for aphid fitness (Vogel and Moran 2011). Results indicated that all aphid sub-lines feeding on fava were of similar mass to one another, but aphid mass was significantly greater in aphid sub-lines feeding on fava compared to alfalfa based on Tukey’s post-hoc tests (χ^2^ = 567.017, d.f. = 1, *P* < 0.0005, Figure 1A). A significant aphid line effect was found for host-plant treatment (χ^2^ = 48.005, d.f. = 1, *P* < 0.0005). One aphid sub-line feeding on alfalfa (A2) was significantly greater in mass compared to the other aphid sub-lines (Figure 1A).

Nutritional endosymbionts, such as *Buchnera*, can be regulated in insects at the bacteriocyte and/or symbiont titer level between different insect lifestages and morphs (Humphreys and Douglas 1997; Mira and Moran 2002; Kono *et al.* 2008; Nishikori *et al.* 2009; Stoll *et al.* 2010; Vigneron *et al.* 2014; Parkinson *et al.* 2016; Simonet *et al.* 2016). To determine if host-plant diet affects the number of aphid bacteriocytes and/or *Buchnera* titer we counted bacteriocytes and *Buchnera* cells and then compared them between host-plant treatments. The numbers of bacteriocytes in 4^th^ instar aphids were not significantly different between host-plant treatments or aphid lines (χ^2^ = 3.522, d.f.=1, *P* = 0.061; χ^2^ = 2.065, d.f.=4, *P* = 0.724, respectively). Average bacteriocyte number per one aphid individual was 68 (95% C.I. 63.8-71.1, N = 45) and 63 (95% C.I. 58.9-66.2, N = 46) for the fava and alfalfa treatments, respectively. Moreover, *Buchnera* abundance did not significantly differ between host-plant treatments based on RT-qPCR (χ^2^ = 2.674, d.f.=1, *P* = 0.102, Figure 1B). Normalized *Buchnera* abundance was higher in first day nymphs compared to first day adults regardless of host-plant treatment (χ^2^ = 55.486, d.f.=1, *P* < 0.0005, Figure 1B). Consistent with these findings, the number of *Buchnera* cells within bacteriocytes did not differ significantly between aphids from representative host-plant treatments based on TEM images (T = 1.275, d.f.=9, *P* = 0.234). Within a unit area (100 µm^2^), an average of 21 (N = 7) and 24 (N = 5) *Buchnera* cells were identified within bacteriocytes of 4^th^ instar fava and alfalfa feeding aphids, respectively (Figure 1C).

### Effects of host-plant diet on the expression of key aphid symbiosis genes

To investigate if host-plant diet affects the expression of key symbiotic genes of aphids at the mRNA level in bacteriocytes, we first conducted RNA-Seq on bacteriocytes and other aphid body tissues (BAC *vs.* BODY) (Table S1). For the BAC *vs.* BODY comparison we identified 1,904 genes that were significantly up-regulated between bacteriocytes and body cells for both host-plant treatments (Dataset S1, Table S2). We further identified 4,211 genes that were significantly down-regulated between bacteriocytes and body cells for both host-plant treatments (Dataset S2, Table S2). To determine host-plant differences between bacteriocytes (ABAC *vs.* FBAC) we identified 54 genes that were up-regulated and 101 genes that were down-regulated in bacteriocytes of alfalfa feeding aphids compared to bacteriocytes of fava feeding aphids (Dataset S3, Table S2).

To characterize the functions of genes differentially expressed in bacteriocytes for each comparison we used GSEA (Subramanian *et al.* 2005). For the BAC *vs.* BODY comparison we found 10 KEGG pathways significantly enriched in bacteriocytes compared to body cells for both host-plant treatments (Table S3a). The top five KEGG pathways in descending order based on the GSEA enrichment score were glycine, serine and threonine metabolism, glyoxylate and dicarboxylate metabolism, phenylalanine metabolism, pentose phosphate pathway, and the nicotinate and nicotinamide metabolism (Table S3a). We also identified five KEGG pathways that were significantly down-regulated in bacteriocytes compared to body cells for both host-plant treatments (Table S3a). Such KEGG pathways in descending order based on the GSEA enrichment score were hippo signaling pathway, notch signaling pathway, other glycan degradation, phototransduction, and neuroactive ligand-receptor interaction.

To identify the pathways that were differentially expressed in bacteriocytes between host-plant treatments (ABAC *vs.* FBAC) we performed GSEA and identified seven KEGG pathways that were significantly enriched in bacteriocytes of alfalfa compared to fava treated aphids. The seven KEGG pathways in descending order based on the GSEA enrichment score were synthesis and degradation of ketone bodies, vitamin B6 metabolism, aminoacyl-tRNA biosynthesis, pyruvate metabolism, Jak-STAT signaling pathway, lipoic acid metabolism, and the butanoate metabolism (Table S3b).

**Figure 2:**
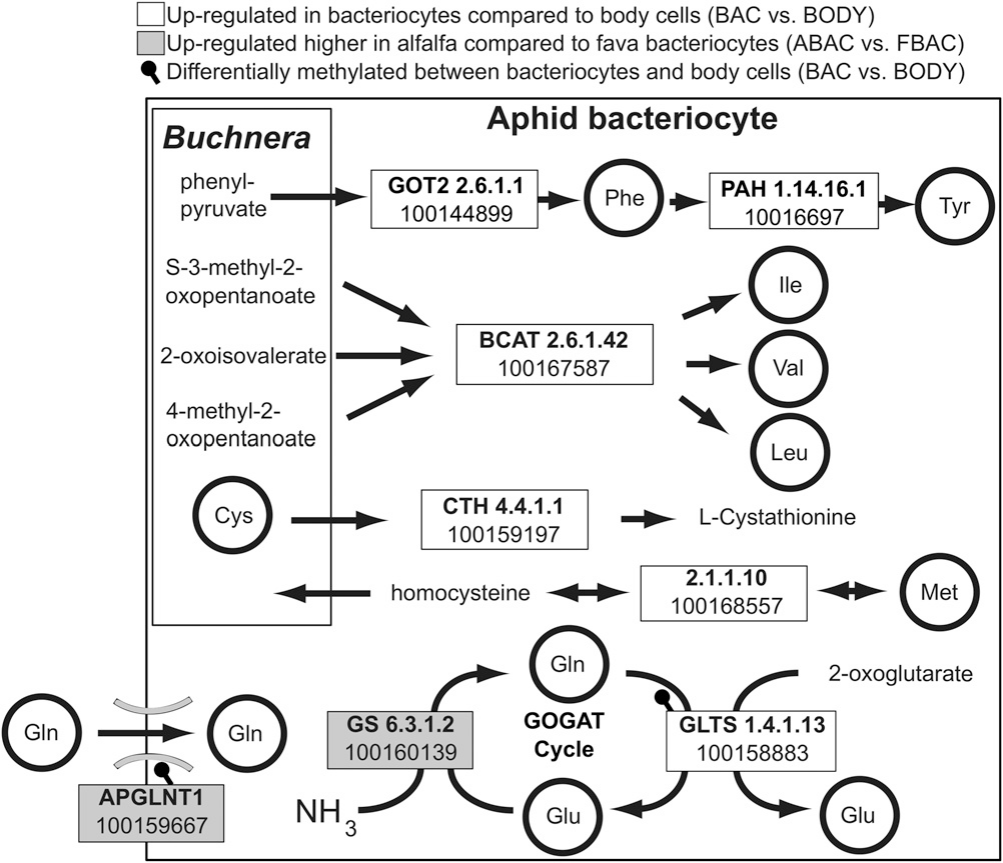
Host-plant effects on differential expression and methylation of key aphid genes that complement and regulate the integrated aphid-*Buchnera* amino acid metabolism. Gene boxes are annotated with either E.C. numbers, Genbank LOC numbers, and/or gene names. Genes are significantly up-regulated in bacteriocytes compared to body cells (BAC *vs.* BODY) and enriched in alfalfa compared to fava bacteriocytes (ABAC *vs.* FBAC) if adjusted p-value ≤ 0.05 and normalized read counts are 50% higher. Genes are differentially methylated significantly if there was ≥10% difference in percent methylation and FDR corrected p-value ≤ 0.01.

Aphid and *Buchnera* metabolisms are integrated for the production of amino acids within bacteriocytes. This shared amino acid metabolism is hypothesized to be regulated by the aphid host via transporters, the GS/GOGAT cycle, and genes that complement *Buchnera*’s essential amino acid pathways (Nakabachi *et al.* 2005; Hansen and Moran 2011; Poliakov *et al.* 2011; Price *et al.* 2014). We examined these aphid genes and found that 22 out of 27 genes were significantly enriched in bacteriocytes relative to body cells for both host-plant treatments (BAC *vs.* BODY; Figure 2). We further identified that two out of eight of these genes (Glutamine synthetase and *ApGLNT1*) were significantly enriched in bacteriocytes of alfalfa feeding aphids when compared to bacteriocytes of fava feeding aphids (ABAC *vs.* FBAC; Figure 2; Dataset S4). Glutamine synthetase (*GS*) is a key enzyme of the GS/GOGAT cycle and recycles ammonia into glutamine (Hansen and Moran 2011). The transporter *ApGLNT1* imports glutamine into bacteriocytes (Price *et al.* 2014). Collectively these results suggest that aphid genes that synthesize and transport glutamine, an important amino donor for *Buchnera*’s essential amino acid pathways, is enriched in bacteriocytes of aphids feeding on their specialized host-plant, alfalfa, compared to their universal host-plant, fava (Figure 2).

**Figure 3:**
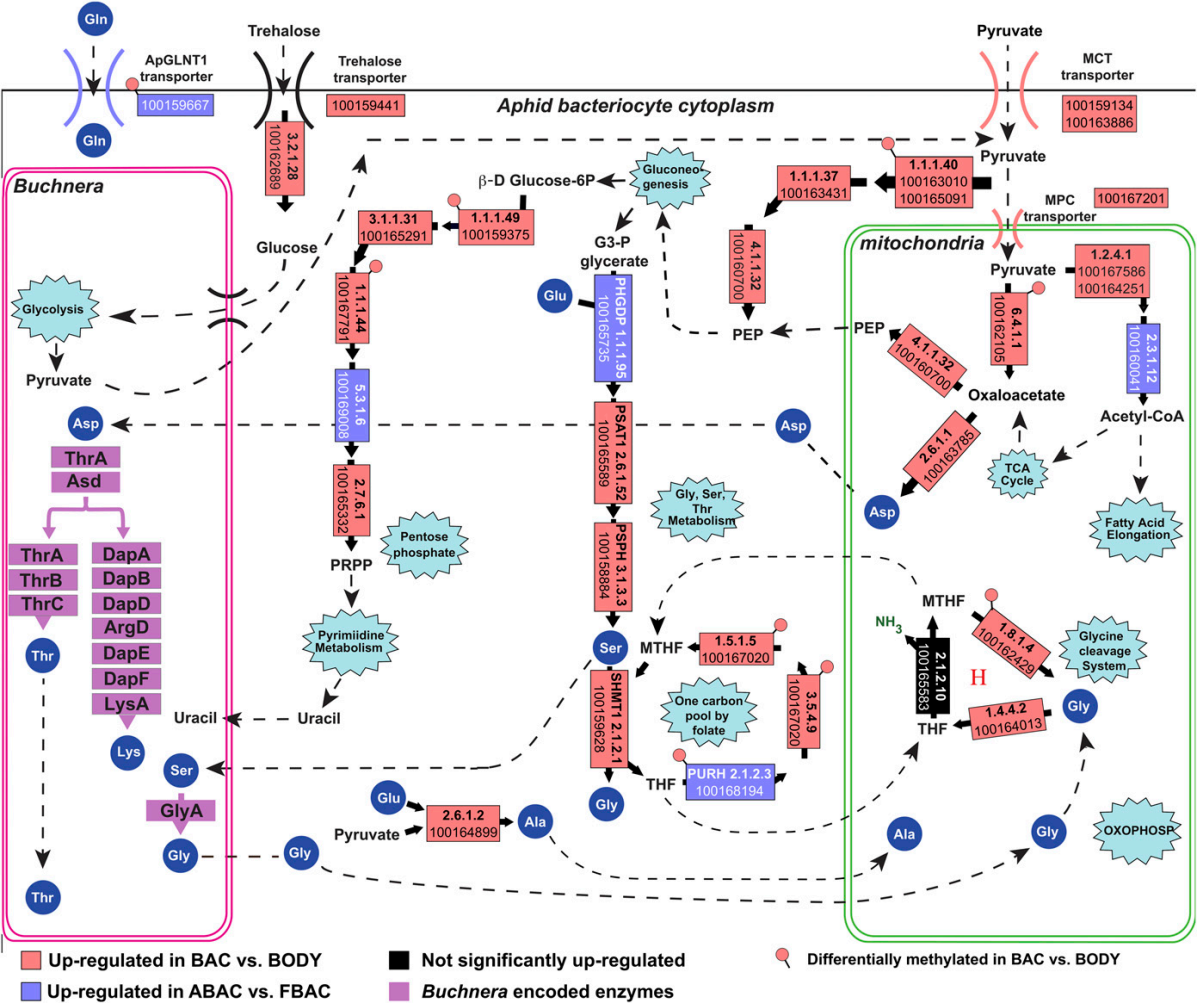
Differential gene expression and methylation of the Glycine/Serine metabolism in aphid bacteriocytes. Gene boxes are annotated with either E.C. numbers, Genbank LOC numbers, and/or gene names. ’*H*’ denotes the glycine cleavage system H protein (LOC100169052). Genes are significantly up-regulated in bacteriocytes compared to body cells (BAC *vs.* BODY) and in alfalfa compared to fava bacteriocytes (ABAC *vs.* FBAC) if adjusted p-value ≤0.05 and normalized read counts are 50% higher. Genes are differentially methylated significantly if there was ≥10% difference in percent methylation and FDR corrected p-value ≤ 0.01.

Another major subset of genes that were enriched significantly in bacteriocytes compared to other body cells for both host-plant treatments (BAC *vs.* BODY) belonged to the glycine/serine metabolism (Figure 3, Dataset S4). Specifically, the genes for serine biosynthesis (D-3-phosphoglycerate dehydrogenase; *PHGDP*, phosphoserine aminotransferase 1; *PSAT1*, phosphoserine phosphatase; *PSPH*) were up-regulated significantly in bacteriocytes compared to body cells. Also, serine hydroxymethyltransferase (*SHMT*), which converts serine to glycine, and the bifunctional purine biosynthesis protein (*PURH*) were up-regulated significantly higher in bacteriocytes compared to body cells. The glycine/serine metabolism also relies on the maintenance of the cofactor tetrahydrofolate (THF). In the bacteriocytes of both treatments THF was maintained by the significant up-regulation of genes in the one carbon pool by folate metabolism, and the production of 5,10-methenyl-THF through the Glycine Cleavage System (Figure 3, Dataset S4). Among such genes, *PHGDP* and *PURH* were significantly higher in bacteriocytes of alfalfa compared to fava feeding aphids (ABAC *vs.* FBAC; Figure 3).

For both host-plant treatments (BAC *vs.* BODY), gluconeogenesis instead of glycolysis appears to be occurring in bacteriocytes compared to body cells as indicated by the up-regulation of malate dehydrogenase (*MDH*) and phosphoenolpyruvate carboxykinase (*PEPCK*) (Figure 3). In turn, instead of glucose, alternative energy substrates such as extracellular and *Buchnera* derived pyruvate may provide the carbon backbone to fuel the glycine/serine metabolism. For example, pyruvate transporters were significantly up-regulated in bacteriocyte cells compared to body cells (monocarboxylate transporter; *MCT* and mitochondrial pyruvate carrier; *MPC*) (Figure 3).

Another key pathway that is important in the aphid-*Buchnera* symbiosis involves the production of uracil. *Buchnera* is unable to produce its own uracil (Shigenobu *et al.* 2000) and therefore it depends on the host for uracil biosynthesis in this integrated metabolism. The uracil salvage pathway, especially pseudouridine kinase, was found to be significantly enriched in bacteriocytes of aphids feeding on alfalfa compared to fava (ABAC *vs.* FBAC; Dataset S4).

### Effects of host-plant diet on DNA methylation profiles

**Figure 4:**
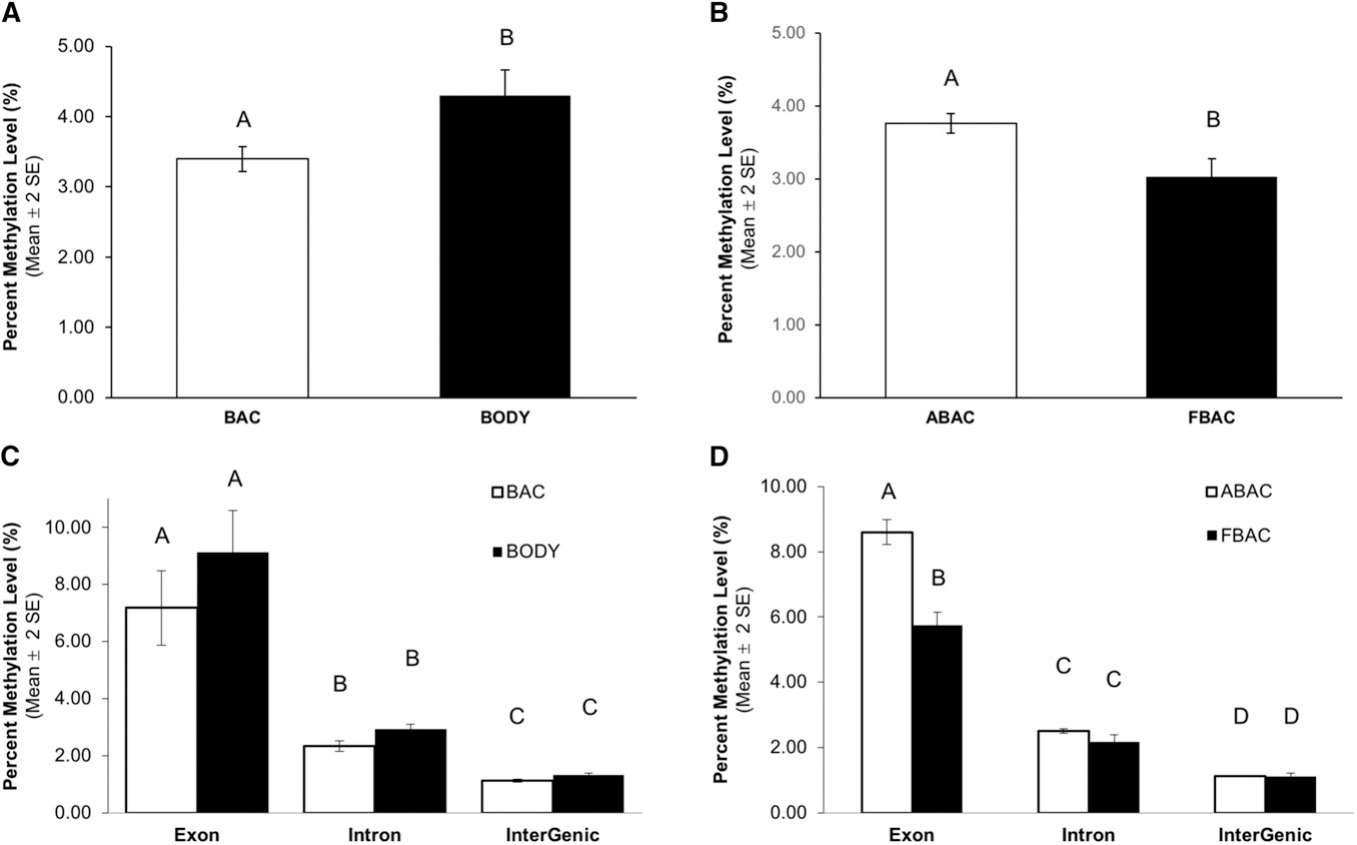
Percent CpG methylation level for bacteriocyte and body samples from each host-plant treatment. Different letters on bars indicate significant difference between each group and within each subfigure (A-D) (paired *t*-test *P* < 0.05). BAC and BODY denote bacteriocytes and body cells, respectively. Each sample has 6 biological replicates from both alfalfa and fava feeding aphids. ABAC and FBAC denote bacteriocytes of alfalfa feeding aphids and fava feeding aphids, respectively. Each sample has 3 biological replicates. (A) Average methylation levels of BAC and BODY. (B) Average methylation levels of ABAC and FBAC. (C) Average methylation levels of genic regions of BAC and BODY. (D) Average methylation levels of genic regions of ABAC and FBAC.

Diet cues can alter DNA methylation patterns within and adjacent to invertebrate genes (Kucharski *et al.* 2008). Moreover, these DNA methylation marks have been associated with active genes and alternative splicing (Yan *et al.* 2015). To determine if host-plant diet affects the methylation of key symbiotic genes in aphid bacteriocytes we used whole-genome bisulfite sequencing (Table S4). The average percentages of CpG methylation were 3.4% and 4.3% for bacteriocytes and body cells, respectively (Figure 4A). Bacteriocytes had a significantly lower percent of CpG methylation compared to body cells (BAC *vs.* BODY; paired *t*-test, t = 13.47, df = 5, *P* < 0.0001) (Figure 4A). The average percentages of CpG methylation were 3.8% and 3.0% for bacteriocytes of alfalfa compared to fava feeding aphids, respectively (Figure 4B). Bacteriocytes of alfalfa feeding aphids had a significantly higher percent of CpG methylation compared to bacteriocytes of fava feeding aphids (ABAC *vs.* FBAC; t = 5.18, df = 4, *P* = 0.0066) (Figure 4B).

To investigate if there was a difference in percent methylation within and outside genic regions percent methylation within the exon, intron, and the intergenic regions were determined. For both host-plant diets there was not a significant difference in percent methylation between bacteriocyte and body cells for the exon, intron, or the intergenic regions (BAC *vs.* BODY; paired *t*-test, t = 1.76, df = 2, *P* = 0.22) (Figure 4C). In contrast, when comparing between host-plant diets percent methylation in the exon region was significantly higher in bacteriocytes of alfalfa feeding aphids compared to bacteriocytes of fava feeding aphids (ABAC *vs.* FBAC; paired *t*-test, t = 10.34, df = 4, *P* = 0.0005) (Figure 4D). The percent of methylation in the intron and intergenic regions were not significantly different in bacteriocytes of alfalfa feeding aphids compared to bacteriocytes of fava feeding aphids (ABAC *vs.* FBAC; paired *t*-test, t = 2.40, df = 2, *P* = 0.14 for introns; t = 0.57, df = 2, *P* = 0.63 for intergenic) (Figure 4D).

Collectively, for all bacteriocyte and body tissue samples percent methylation within the exon regions was significantly higher compared to the intron regions ((paired *t*-test, t = 12.05, df = 11, *P* < 0.001) (Figure 4C), t = 8.50, df = 5, *P* < 0.001) (Figure 4D)). Also, percent methylation was significantly higher in the intron regions compared to the intergenic regions for all samples ((paired *t*-test, t = 19.03, df = 11, *P* < 0.001), (Figure 4C), (t = 17.02; df = 5; *P* < 0.001) (Figure 4D)).

**Figure 5:**
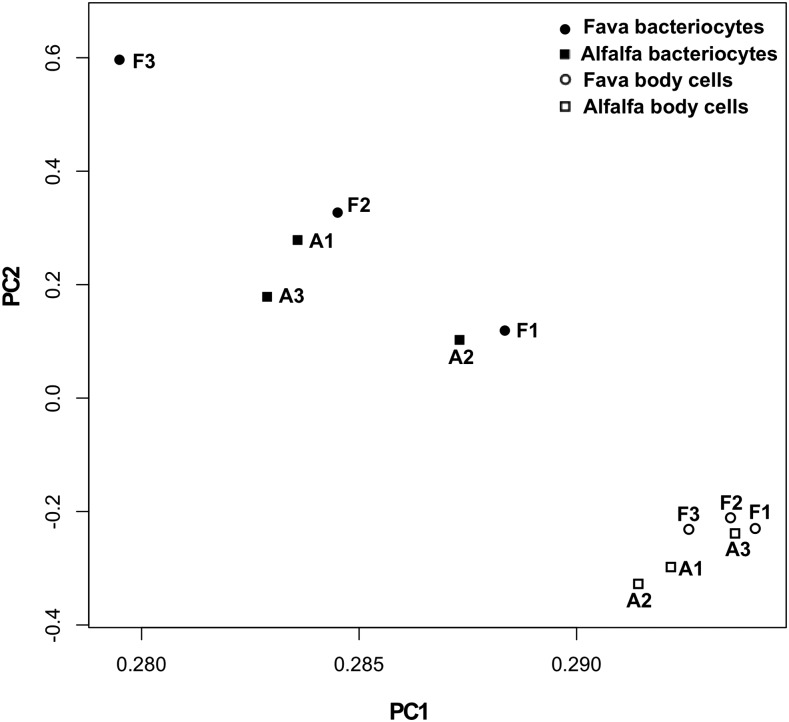
Principal Component Analysis of CpG methylation profiles for each bacteriocytes and body sample. F1, F2, and F3 denote 3 biological replicates of aphid sub-lines within the fava treatment. A1, A2, A3 denote 3 biological replicates of aphid sub-lines within the alfalfa treatment. PC1 explains 93.1% of total variance with standard deviation of 3.34. PC2 explains 1.5% of total variance with standard deviation of 0.43.

In order to visually determine how shared CpG sites differ between all samples in percent methylation when aphids feed on their specialized compared to universal host-plant diet we conducted a Principal Component Analysis (Figure 5). To test if methylated CpG profiles were significantly different between bacteriocyte, body cell, and host-plant treatments we used multi-response permutation procedure (MRPP) (Mielke and Berry 2003). We found that the four *a priori* groups: bacteriocytes of alfalfa feeding aphids (ABAC), body cells of alfalfa feeding aphids (ABODY), bacteriocytes of fava feeding aphids (FBAC), and body cells of fava feeding aphids (FBODY) were significantly different from one another in CpG methylation profiles (*P* < 0.001; A = 0.09356). In the PCA ordination, all body samples clustered tightly together in ordination space away from bacteriocyte samples. Using MRPP, we found that methylated CpG profiles of bacteriocyte samples were significantly different compared to body cell samples (BAC *vs.* BODY; *P* < 0.005; A = 0.08245), with a significantly higher dispersion of within-group differences for bacteriocyte samples (delta = 0.2759) compared to body cell samples (delta = 0.1772). In accord to the PCA ordination MRPP results indicate that bacteriocyte cells have significantly different methylation profiles compared to body cells, which have more similar distributions to one another, regardless of host-plant treatment (Figure 5). Nevertheless, body cells are a mixture of different aphid cell types and therefore we cannot exclude the possibility that more abundant host cell types mask host-plant differences of less abundant cell types. In contrast, among bacteriocyte samples (ABAC *vs.* FBAC), fava samples are more heterogeneous in CpG profiles compared to alfalfa samples, with a higher dispersion of within-group differences for FBAC (delta = 0.2810) compared to ABAC (delta = 0.2645) (Figure 5). These results indicate that methylation profiles are specific to aphid cell type and host-plant diet, especially for bacteriocytes in the aphid’s specialized host-plant treatment, alfalfa.

A total of 3,474 CpG sites were significantly differentially methylated between bacteriocytes and body cells of both host-plant treatments (BAC *vs.* BODY) (Table S5a). Between bacteriocytes of alfalfa feeding aphids compared to fava feeding aphids a total of 294 CpG sites were differentially methylated significantly (ABAC *vs.* FBAC). For both comparisons, differential CpG methylation was primarily confined to the gene body regions (82% for BAC *vs.* BODY; 78% for ABAC *vs.* FBAC) (Table S5b) as revealed in other non-mammal animals, which may contribute to gene activation and/or alternative splicing (Hunt *et al.* 2013).

To link the patterns of differential DNA methylation with differential gene expression, we identified 441 genes that were both differentially methylated and differentially expressed significantly between bacteriocyte and body samples for both alfalfa and fava feeding aphids (BAC *vs.* BODY) (Table S5). All 441 genes were up-regulated and hypomethylated in bacteriocytes relative to body cells. We also identified 702 genes that are both differentially methylated and differentially spliced between bacteriocyte and body samples of both alfalfa and fava feeding aphids (BAC *vs.* BODY) (Table S5; Dataset S4). Furthermore, we identified three genes that were both differentially methylated and differentially expressed significantly between bacteriocytes of alfalfa compared to fava feeding aphids (ABAC *vs.* FBAC; Table S5). All three genes were down-regulated and hyper-methylated in alfalfa compared to fava feeding aphid bacteriocytes. These genes were the serine/arginine repetitive matrix 1 gene (LOC100160294), the proton-coupled amino acid transporter 4-like (LOC100159667), and the broad-complex core protein isoforms 1/2/3/4/5-like gene (LOC100167015). We also identified three genes that were both differentially methylated and differentially spliced between bacteriocyte samples from alfalfa and fava feeding aphids (ABAC *vs.* FBAC; Table S5): anoctamin-1-like (LOC100167803), formin-binding protein 1-like (LOC100166693), and tumor protein D54-like (LOC100164449).

Using GSEA (Subramanian *et al.* 2005), we found five pathways that were both significantly differentially methylated (hypo-methylated) and up-regulated in bacteriocytes compared to body cells. These five pathways were the metabolic pathways, lysosome, protein processing in endoplasmic reticulum, selenocompound metabolism, and tryptophan metabolism (Table S3c). No pathways were differentially methylated and significantly down-regulated in bacteriocytes compared to body cells (BAC *vs.* BODY; Table S3c). Genes that were both differentially methylated and differentially spliced between bacteriocyte and body samples (BAC *vs.* BODY) belong to 16 KEGG pathways (Table S6). These pathways include the bulk movement into cells and digestion (15 genes), the degradation, processing, and transport of RNAs (47 genes), four different signaling pathways including one involved in the immune response (Jak-Stat) (37 genes), protein processing and degradation (20 genes), and the biosynthesis of amino acids (6 genes) (Figure 2).

Fourteen key aphid genes associated with the integrated aphid-*Buchnera* symbiosis and the glycine/serine metabolism were both differentially expressed and differentially methylated between the bacteriocytes and body cells (BAC *vs.* BODY) (Figures 2, 3; Dataset S4). For example, glutamate synthase, an important enzyme of the GS/GOGAT cycle that converts glutamine to glutamate was both hypo-methylated, differentially spliced, and up-regulated in bacteriocytes of both fava and alfalfa feeding aphids compared to body cells (Figure 3). In addition, an active glutamine transporter (*ApGLNT1*) that was previously characterized to be important for the regulation of *Buchnera*’s essential amino acid biosynthesis pathways (Price *et al.* 2014) was significantly hypo-methylated and up-regulated in bacteriocytes compared to body cells (Figure 2). Also, two enzymes (*PURH* and cytoplasmic C-1-tetrahydrofolate synthase) in the one carbon pool by folate pathway were hypo-methylated and up-regulated in the bacteriocytes compared to the body cells (Figure 3).

## Discussion

In this study, we demonstrated for the first time that key aphid genes involved in the regulation of the aphid-*Buchnera* symbiosis are differentially expressed, spliced, and methylated when aphids feed on a specialized compared to a universal host-plant diet. Our data indicate that this regulatory and epigenetic response to distinct host-plant types that vary in amino acid profiles may play a significant role in modulating the aphid-*Buchnera* amino acid metabolism when aphids feed on their specialized compared to universal host-plant diet. Moreover, this regulatory response in combination with lower aphid fitness when aphids feed on their specialized compared to universal host-plant diet is consistent with the pea aphid engaging in a compensatory metabolic response when it specializes on a less suitable host-plant. We also identified key aphid genes and pathways involved in the aphid-*Buchnera* symbiosis that are differentially expressed, spliced, and methylated in both host-plant diets. These results collectively suggest that DNA methylation may play both a conserved (maintenance methylation) and an environmentally induced (*de novo* methylation) regulatory role in bacteriocytes when aphids feed on host-plant diets that vary in amino acid profiles.

Here we reveal that instead of regulating bacteriocyte or *Buchnera* cell number the aphid-*Buchnera* integrative metabolism modulates patterns of bacteriocyte DNA methylation and gene expression in response to its specialized host-plant diet, alfalfa, when compared to its universal host-plant diet, fava. Results from our study that provide evidence for this finding include the following: ***1)*** One aphid enzyme (Glutamine synthetase; *GS*) in the GS/GOGAT cycle was enriched in bacteriocytes of alfalfa feeding aphids compared to fava feeding aphids (Figure 2). The GS/GOGAT cycle is hypothesized to play a key role with *Buchnera* in sustaining aphids on a nitrogen-limited diet, because *GS* recycles waste ammonia for the production of glutamine (Hansen and Moran 2011). ***2)*** The transporter *ApGLNT1* is significantly up-regulated in the bacteriocytes of alfalfa feeding aphids compared to fava feeding aphids (Figure 2). Interestingly this transporter was significantly hypo-methylated only in bacteriocytes compared to body cells of both alfalfa and fava feeding aphids. This transporter imports glutamine into bacteriocytes and is inhibited by arginine produced by *Buchnera*. In turn, this transporter may play a key role in regulating *Buchnera*’s essential amino acid metabolism (Price *et al.* 2014), by increasing essential amino acid biosynthesis in alfalfa feeding aphids**. *3)*** The vitamin B6 pathway was significantly enriched in bacteriocytes of alfalfa feeding aphids compared to fava feeding aphids (Table S3b). Vitamin B6 is an essential cofactor in animals and microbes and plays an important role in the amino acid and carbohydrate metabolism and singlet oxygen resistance (John 1995; Daub and Ehrenshaft 2000). Collectively, both the aphid and *Buchnera* do not encode the entire vitamin B6 biosynthesis I or II pathways, however *Buchnera* still encodes serC and thrC and the aphid encodes the enzymes 2.6.1.52, 2.7.1.35, and 1.4.3.5., which make up the majority of the pathway. Both enzymes 2.6.1.52 and 2.7.1.35 are up-regulated significantly in bacteriocytes compared to body tissues 15X and 8X respectively suggesting that there is a demand for vitamin B6 biosynthesis in aphid bacteriocytes. ***4)*** The uracil salvage pathway was found to be significantly enriched in bacteriocytes of aphids feeding on alfalfa compared to fava. These results suggest that more uracil potentially for both *Buchnera* and/or aphid mRNA biosynthesis is needed for aphid bacteriocytes when they feed on alfalfa compared to fava. ***5)*** Also six genes were both differentially expressed/spliced and methylated between bacteriocytes of alfalfa compared to fava bacteriocytes and are involved in amino acid transport, protein kinase activity, calcium activated chloride channel activity, gene regulation via epigenomic interactions utilizing the POZ zinc finger domain, and unknown function. More information on how these genes may help regulate the integrated aphid-*Buchnera* metabolism when aphids feed on different host-plants is needed. Collectively, these results suggest that when aphids feed on their suboptimal, specialized host-plant, alfalfa, key aphid genes involved in the regulation of the integrative amino acid metabolism are enriched in bacteriocytes.

The observed regulatory changes in bacteriocytes are indicative of a compensatory response of aphids feeding on alfalfa that require more essential amino acids compared to aphids on fava. For example, previous amino acid concentration data shows a limitation of essential amino acids in alfalfa sap compared to fava sap (Sandström and Pettersson 1994). Specifically, Sandström and Pettersson (1994) revealed that total amino acid concentrations in sap obtained from aphid stylets are relatively similar between alfalfa and fava, however concentrations of nine essential amino acids (arginine, isoleucine, leucine, lysine, phenylalanine, tryptophan, tyrosine, valine, and histidine) are lower in alfalfa sap compared to fava sap. Our data coincides with these amino acid concentration data (Sandström and Pettersson 1994), because we revealed an up-regulation of key aphid genes and pathways that are important in provisioning amino donors, co-factors, and energy to fuel the integrative amino acid metabolism with *Buchnera* for the production of essential amino acids.

In this study, we also observed that regardless of host-plant treatment 1,143 genes were differentially expressed/spliced and methylated in aphid bacteriocytes compared to body cells. These genes included glutamate synthase, the other gene involved in the GS/GOGAT cycle (Figure 2), as well as several pathways involved in cell signaling, immune function, and the regulation of RNA and protein biosynthesis and degradation. One of the most distinctive and highly enriched pathways identified within bacteriocytes compared to body cells was the glycine/serine metabolic profile (Figure 3). Interestingly, to the best of our knowledge this metabolic profile has only been identified previously as a metabolic hallmark in cancer cells (Perroud *et al.* 2006; Jain *et al.* 2012; Amelio *et al.* 2014; Locasale 2013). Unlike some cancer cells, however, expression profiles of bacteriocytes in our study reveal that gluconeogenesis instead of glycolysis is occurring (Figure 3); *i.e.*, the Warburg effect, where cellular energy production relies heavily on aerobic glycolysis (Vander Heiden *et al.* 2009), is not occurring. This pattern of carbohydrate utilization in pea aphid bacteriocytes was identified previously (Poliakov *et al.* 2011). An alternative energy source instead of glucose, such as pyruvate, may be metabolized to fuel the glycine/serine metabolism. This model of using pyruvate as the carbon skeleton for the glycine/serine metabolism has been proposed previously in breast cancer cells (Diers *et al.* 2012). Similar to breast cancer cells we also observed the up-regulation of the pyruvate transporters MCT and MPC and the enzymes that utilize the carbon skeleton of pyruvate into gluconeogenesis in bacteriocyte cells (Figure 3). Currently, it is unclear why cancer cells display this distinctive glycine/serine enriched metabolic profile, however several hypotheses have been proposed, such as reducing oxidative stress from cells that are metabolically very active (Jain *et al.* 2012; di Salvo *et al.* 2013; Maddocks *et al.* 2013; Labuschagne *et al.* 2014). Investigating the regulatory mechanisms behind the differences between metabolically active and prolific cancer cells and metabolically active, yet not prolific aphid bacteriocytes can become a key factor in elucidating the significance of how and why the glycine metabolism is adopted in both malignant cancer cells and symbiotic cells.

The genome-wide CpG methylation patterns of the pea aphid showed higher levels of methylation in gene bodies, especially in alfalfa feeding bacteriocytes (Figure 4B), which is consistent with methylation patterns of other insect species with functional methylation systems (Suzuki *et al.* 2007; Feng *et al.* 2010; Zemach *et al.* 2010; Provataris *et al.* 2018). Bacteriocytes from both host-plant treatments revealed significantly lower percent CpG methylation levels compared to the body cells of pea aphid (Figure 4A). These results suggest that more exon and intron regions within bacteriocytes are hypo-methylated compared to body cells. Host-plant treatments also influenced hypo- *vs.* hyper-methylation patterns in our study, as hundreds of genes were differentially methylated between bacteriocytes and body cells and between bacteriocytes depending on what host-plant they fed upon. In one study (Huh *et al.* 2013) it has been hypothesized that methylation inside of gene bodies plays an important role in reducing transcriptional noise (Huh *et al.* 2013). Differential methylation within gene bodies has also been associated with alternative gene splicing (Shukla *et al.* 2011). Here we observed hundreds of genes that were both differentially spliced and methylated within bacteriocytes compared to body cells and between bacteriocytes of alfalfa compared to fava feeding aphids. Genes that are hyper-methylated may also result in higher gene expression levels in insects. For example, a recent study suggested that gene body methylation of the lysosomal alpha-mannosidase (*LAM*) gene of *Apis melifera* increased the expression level of *LAM* (Wedd *et al.* 2016). In contrast to this latter study, all genes that were both differentially expressed and methylated in this current study were hypo-methylated and up-regulated, which is more in line with what is observed in vertebrates but within the promoter regions (Jones 2012).

The effect of host-plant treatment on DNA methylation patterns within pea aphid bacteriocytes was evident in our study. For example, our results reveal that the CpG methylation profiles of fava feeding aphid bacteriocytes were more heterogeneous between biological replicates compared to alfalfa feeding bacteriocytes (Figure 5). Such heterogeneity may have come from the relaxed nutritional constraints of the ‘universal’ host-plant, fava, compared to the specialized host-plant, alfalfa. Alternatively, environmental cues from its specialized host-plant, alfalfa, may induce host-plant specific methylation profiles. In this study we identified gene candidates to examine further that were both differentially expressed/spliced and methylated in bacteriocytes compared to body cells and between bacteriocytes when aphids fed on different host plant diets. If host-plant specific patterns of DNA methylation induce tissue and host-plant specific gene expression profiles in bacteriocytes this may be a key regulatory factor that induces phenotypic variation of the integrative metabolism in response to host-plant diet.

In summary, our findings indicate that when aphids feed on their specialized host-plant, alfalfa, key aphid genes that are involved in the integrative metabolism with *Buchnera* are differentially expressed/spliced, and some of these are differentially methylated. Future studies are needed to investigate how and if these methylated sites can influence the regulation of the bacteriocyte, be inherited, and ultimately drive host-plant specialization broadly in sap-feeding insect-nutritional symbioses. We hypothesize that this host-plant induced metabolic modification to the aphid’s integrative metabolism may ultimately allow aphids to utilize host-plant diets that were once unsuitable.
